# miRNA, transcriptional regulation and cognitive decline

**DOI:** 10.1002/alz.083400

**Published:** 2025-01-03

**Authors:** Andre Fischer, Farahnaz Sananbenesi, Kwangsik Nho, Dennis Manfred Krüger, Leslie M. Shaw, Andrew J. Saykin, Ivana Delalle

**Affiliations:** ^1^ German Center For Neurodegenerative Diseases (dzne), Goettingen, Lower Saxony Germany; ^2^ German Center For Neurodegenerative Diseases (dzne), Goettingen Germany; ^3^ Indiana University School of Informatics and Computing, Indianpolis, IN USA; ^4^ Center for Computational Biology and Bioinformatics, Indiana University School of Medicine, Indianapolis, IN USA; ^5^ Department of Radiology and Imaging Sciences, Indiana University School of Medicine, Indianapolis, IN USA; ^6^ German Centre for Neurodegenerative Diseases (DZNE), Göttingen, Lower Saxony Germany; ^7^ Center for Neurodegenerative Disease Research, University of Pennsylvania, Philadelphia, PA USA; ^8^ Department of Pathology and Laboratory Medicine, Philadelphia, PA USA; ^9^ Department of Pathology and Laboratory Medicine, Perelman School of Medicine, University of Pennsylvania, Philadelphia, PA USA; ^10^ University of Pennsylvania, Philadelphia, PA USA; ^11^ Dept of Pathology & Laboratory Medicine, University of Pennsylvania, Perelman School of Medicine, Philadelphia, PA USA; ^12^ Indiana Alzheimer’s Disease Research Center, Indianapolis, IN USA; ^13^ Department of Medical and Molecular Genetics, Indiana University School of Medicine, Indianapolis, IN USA; ^14^ Indiana University, Indianapolis, IN USA; ^15^ Center for Neuroimaging, Department of Radiology and Imaging Sciences, Indiana University School of Medicine, Indianapolis, IN USA; ^16^ Indiana University School of Medicine, Indianapolis, IN USA; ^17^ Boston University, Boston, MA USA

## Abstract

**Background:**

Despite significant advancements in the development of blood biomarkers for AD, challenges persist due to the complex interplay of genetic and environmental risk factors in AD pathogenesis. Epigenetic processes, including non‐coding RNAs and especially microRNAs (miRs), have emerged as important players in the molecular mechanisms underlying neurodegenerative diseases. MiRs have the ability to fine‐tune gene expression and proteostasis, and microRNAome profiling in liquid biopsies is gaining increasing interest since changes in miR levels can indicate the presence of multiple pathologies. We have profiled blood samples via smallRNA sequencing for 1056 individuals of the DELCODE and 847 individuals of the ANDI cohort.

**Methods:**

We profiled blood samples via smallRNA sequencing for 1056 individuals of the DELCODE (German Longitudinal Cognitive Impairment and Dementia Study) and 847 individuals of the ANDI (Aging and Dementia in the Community) cohort, consisting of individuals diagnosed with SCD, MCI, AD, or control.

**Results:**

By applying differential expression, WGCNA, as well as linear and non‐linear machine learning approaches, we identify microRNA signatures that can help identify patients at distinct stages of disease progression, as well as signatures that can predict the course of the disease. These data are compared with phenotyping data, such as cognitive function and ATN biomarkers. We will also discuss the role of other non‐coding RNAs besides microRNAs and provide a framework for developing RNA‐based point‐of‐care assays.

